# Influence of Immunogenicity on the Efficacy of Long-Term Treatment with TNF***α*** Blockers in Rheumatoid Arthritis and Spondyloarthritis Patients

**DOI:** 10.1155/2015/604872

**Published:** 2015-04-27

**Authors:** Inesa Arstikyte, Giedre Kapleryte, Irena Butrimiene, Algirdas Venalis

**Affiliations:** ^1^Centre of Rheumatology, Vilnius University, Santariskiu Street 2, LT-08661 Vilnius, Lithuania; ^2^State Research Institute, Centre for Innovative Medicine, Zygimantu Street 9, LT-01102 Vilnius, Lithuania

## Abstract

*Objective*. To analyze the clinical relevance of the levels of TNF*α* blockers and anti-drug antibodies (anti-drug Ab) in patients with rheumatoid arthritis (RA) and spondyloarthritis (SpA) treated with adalimumab (ADA), etanercept (ETA), or infliximab (INF) for a prolonged period of time. *Methods*. Clinical characteristics (disease activity, and adverse events), serum TNF*α* blockers, and anti-drug Ab levels were evaluated in 62 RA and 81 SpA patients treated with TNF*α* blockers for a median of 28 months. *Results*. Anti-ADA Ab were detected in 1 (4.0%) and anti-INF Ab in 14 out of 57 (24.6%) RA and SpA patients. Patient with anti-ADA Ab and 57.1% patients with anti-INF Ab were considered nonresponders to treatment. Anti-ETA Ab were not found in any of 61 ETA treated patients. Anti-ADA and anti-INF Ab levels differ between responders and nonresponders (*P* > 0.05). Three (5.3%) patients with high serum anti-INF Ab levels developed infusion related reactions. Patients with anti-INF Ab more often required changing to another biologic drug (OR 11.43 (95% CI 1.08–120.93)) and treatment discontinuation (OR 9.28 (95% CI 1.64–52.52)). *Conclusion*. Patients not responding to treatment had higher serum anti-ADA and anti-INF Ab concentrations. Anti-INF Ab formation is related to increased risk of infusion related reactions, changing to another biologic drug, and treatment discontinuation.

## 1. Introduction

Tumor necrosis factor alpha (TNF*α*) blockers, such as adalimumab (ADA), etanercept (ETA), and infliximab (IFX), are playing a significant role in the treatment of autoimmune inflammatory diseases such as rheumatoid arthritis (RA) and spondyloarthritis (SpA). Unfortunately, about one-third of patients do not respond to treatment with TNF*α* blockers. For some patients it is due to primary treatment failure (medication is ineffective or serious side effects appear) or due to secondary treatment failure when TNF*α* blocker loses its effectiveness after an initial good response. Previous studies have shown that clinical response in RA patients is related to ADA, ETA, and INF serum levels; while in ankylosing spondylitis (AS) the literature reports controversial data [1–4]. Antibody (Ab) formation leads to a lower TNF*α* blocker concentration [5]. This is explained by immune complex formation between biologic medication and Ab with neutralization of the functional part of the drug and an increased clearance of the drug [5]. It is proved in previous studies that anti-drug antibody (anti-drug Ab) levels inversely correlate with therapeutic response and drug levels (one of the reasons for secondary treatment failure) [4–6]. It was demonstrated that only 4% of patients with anti-adalimumab antibodies (anti-ADA Abs) achieve clinical remission compared with 34% anti-ADA Abs negative ones [6]. In many studies anti-etanercept antibodies (anti-ETA Abs) were not detectable or only in a low number of patients and did not impact the clinical response, indicating that ETA is less immunogenic [4, 7–9]. The appearance of antibodies (Abs) against the drug has been described in about half of the patients receiving repeated TNF*α* monotherapy; as a consequence, immune suppression by concomitant administration of methotrexate (MTX) is recommended both in RA and SpA patients [10–18]. Previous studies show that detectable Abs decrease TNF*α* blockers response as much as 80% [19]. ADA, ETA, and INF can induce the formation of Abs, resulting in loss of efficacy and appearance of side effects such as infusion or injection related reactions [8, 20–22].

Most of the studies were made with only one or two biologic medications without comparing differences in patients suffering from different inflammatory diseases. The aim of our study was to assess the relationship between clinical response, adverse events, and TNF*α* blockers serum levels and antidrug Ab concentrations in RA and SpA (AS and psoriatic arthritis (PsA)) patients treated with ADA, ETA, and INF for a long period of time. We present data on 143 RA and SpA patients whose blood samples were collected once during treatment with ADA, ETA, or INF in Centre of Rheumatology from January 2012 to December 2013.

## 2. Patients and Methods

143 patients (62 with RA and 81 with SpA (49 AS and 32 PsA patients), 69 (48.3%) males), receiving treatment with one of TNF*α* blockers (ADA, *n* = 25 (17.4%), ETA, *n* = 61 (42.7%), or INF, *n* = 57 (39.9%)), were included in this analysis. Patient's mean age (±SD) was 44.98 (±13.38) years at the beginning of treatment with TNF*α* blockers. This was a retrospective observational study approved by the local Ethics Committee. Patients signed an informed consent form according to the Declaration of Helsinki. All patients before initiation treatment with one of TNF*α* blocker fulfilled the American College of Rheumatology (ACR) 1987 revised criteria for RA and the Assessment of SpondyloArthritis international Society (ASAS) 2010 criteria for axial and peripheral SpA. Before initiation of TNF*α* blocker treatment all patients had evidence of active disease, as indicated by a Disease Activity score in 28 joints (DAS-28), 5.76 ± 1.35 (mean ± SD) for RA; swollen (10 ± 8) and tender (20 ± 14) joints for SpA (peripheral forms); and ankylosing spondylitis disease activity score (ASDAS) 15.41 ± 6.13 for axial SpA (see Table 1 for patient's characteristics before initiation of TNF*α* blocker therapy). Blood samples were taken from all patients treated with ADA and INF in the centre. The biggest group of patients with TNF*α* blockers in our centre is treated with ETA. In order to have approximately the same number of patients with ETA comparing with ADA and INF, every third patient was selected to analyze blood samples.

Tables 2 and 3 present patient's characteristics at the time the blood samples were collected. At the beginning of treatment with INF all patients received 2.7 (±1.67) mg/kg (2.86 (±1.67) for RA and 2.59 (±1.67) for SPA patients). However, due to an inadequate response to the initial dose in 28 (49.1%) patients a gradual escalation of INF dose to 3.98 (±1.74) mg/kg (3.97 (±0.72) for RA and 3.98 (±2.4) for SpA patients) was given. All patients were treated with ADA 40 mg every 2 weeks and ETA 50 mg/week subcutaneously during the study.

Lower INF doses were given to all TNF*α* blockers naive RA and SpA patients in our centre because previous studies have shown that in part of the patients these doses were effective [23, 24]. In addition, our center obtained similar results in a retrospective study of RA and SpA patients treated with TNF*α* blockers [25]. This allowed us as a country with a comparatively lower gross domestic product to treat those patients with lower doses of INF [26].

We divided patients into those responding to treatment with TNF*α* blockers (responders) and those not responding (nonresponders). RA patients, whose DAS28 was <3.2 or decreased >1.2 since the initial value, were considered as having good EULAR response; DAS28 ≥3.2 but ≤5.1 or decreased ≤1.2 but ≥0.6, moderate EULAR response; DAS28 >5.1 or decreased <0.6, no response to treatment [27]. RA patients with good or moderate EULAR response were considered as responders, others as nonresponders. SpA patients with ASDAS (calculated with CRP) <1.3 were considered as having inactive disease; whose ASDAS was >1.3 but <2.1, moderate disease activity; >2.1 but <3.5, high disease activity; and >3.5, very high disease activity [28]. SpA patients with inactive disease or moderate disease activity were attributed to responders while patients with high or very high disease activity were considered as nonresponders.

Serum samples were collected once (from January 2012 to December 2013) during the treatment course and were stored at −80°C until TNF*α* blocker and anti-drug Ab were measured. Patient's clinical and laboratory data, diagnosis, disease duration, start of the biologic therapy, adverse events, erythrocyte sedimentation rate (ESR) and C reactive protein (CRP), disease activity information, such as visual analogue scales, disease activity score in 28 joints (DAS28), ankylosing spondylitis disease activity score (ASDAS), and health assessment questionnaire (HAQ) were assessed at the same time blood samples for immunogenicity were collected. Patient's blood samples were collected at least after 3 months of treatment with one of the TNF*α* blockers, before dosing the next scheduled dose (ADA and ETA, before scheduled injection and INF, 8 weeks after last dose, just before next scheduled infusion). Blood samples of ADA treated patients were collected at medium 6 months (interquartile range (IQR) 3–18), ETA, 30 months (IQR 12–54) and INF, medium 42 months (IQR 12–66) after the treatment initiation (Table 2).

Blood samples were analyzed in Centre of Laboratory Medicine of Vilnius University, using Promonitor ADA, Promonitor ETA, and Promonitor INF test kits (Progenika, Derio, Spain) [29].

### 2.1. Serum ADA and ETA Assay Principle

Promonitor ADA and Promonitor ETA are a sandwich enzyme-linked immunosorbent assay (ELISA) [21]. The microwell strips are provided precoated with an anti-ADA and anti-ETA human F(ab′)_2_ fragment. Diluted calibrators, controls, and diluted patient samples are added to separate wells, allowing TNF*α* blocker present to bind to preimmobilized anti-drug Ab. Unbound sample is washed away and a second enzyme horseradish peroxidase- (HRP-) labeled anti-drug monoclonal Ab is added to each well. A second incubation step allows the HRP-labeled anti-drug monoclonal Ab to bind to the TNF*α* blocker that has become attached to the microwells. After washing away the excess of unbound HRP-labeled anti-drug Ab, the remaining enzyme activity is measured by adding a chromogenic substrate and measuring the intensity of the color that develops in a spectrophotometer. The signal obtained is proportional to the amount of the drug in the patient sample. ADA concentration ≤0.024 *μ*g/mL and ETA concentration ≤0.035 *μ*g/mL were considered as negative.

### 2.2. Serum INF Assay Principle

Promonitor INF is a capture ELISA [21]. The microwell strips are provided precoated with an anti-INF human F(ab′)_2_ fragment bound to human recombinant TNF*α*. This format ensures that TNF*α* structure is not disrupted and is available to bind to INF. Diluted calibrators, controls, and diluted patient samples are added to separate wells, allowing INF present to bind to preimmobilized TNF*α*. Unbound sample is washed away and a specific HRP-labeled anti-INF monoclonal Ab is added to each well. A second incubation step allows the anti-INF Ab to bind to the INF that has become attached to the microwells. After washing away the excess of unbound HRP-labeled anti-INF Ab, the remaining enzyme activity is measured by adding a chromogenic substrate and measuring the intensity of the color that develops in a spectrophotometer. The signal obtained is proportional to the amount of the drug in the patient sample. INF concentration ≤0.035 *μ*g/mL was considered as negative.

### 2.3. Serum Anti-Drug Ab Assay Principle

Promonitor anti-ADA, Promonitor anti-ETA, and Promonitor anti-INF are bridging ELISA tests [21]. The microwell strips are provided precoated with TNF*α* blocker. The bridging ELISA takes advantage of the two arms of IgG subclasses 1, 2, and 3, to crosslink the TNF*α* blocker coated on the plane. Calibrators, controls, and diluted patient samples are added to separate wells, allowing anti-TNF*α* blocker Ab present to bind to preimmobilized TNF*α* blocker. Unbound sample is washed away and HRP-labeled TNF*α* blocker is added to each well. A second incubation allows the HRP-labeled TNF*α* blocker to bind to the Ab that has become attached to the microwells. After washing away unbound HRP conjugate, the remaining enzyme activity is measured by adding a chromogenic substrate and measuring the intensity of the color that develops in a spectrophotometer. The signal obtained is proportional to the amount of anti-TNF*α* blocker Ab in the patient sample. Anti-ADA Ab concentration ≤3.5 AU/mL, anti-ETA Ab concentration ≤142.0 AU/mL, and anti-INF Ab concentration ≤2.0 AU/mL were considered as negative.

### 2.4. Statistical Analysis

Descriptive statistics were provided using the mean, standard deviation (SD), median (Md), and interquartile range (IQR). Frequency data were compared by the Pearson's chi-square and Fisher's exact tests. Differences in quantitative values between groups were analysed using Mann-Whitney *U* nonparametric test and *P* < 0.05 was considered statistically significant. Statistical analysis was performed using the Statistical Package for the Social Sciences version 17.0 (SPSS, Chicago, IL, USA).

## 3. Results

Anti-ADA Ab were detected in one patient (4.0%) with undetectable serum ADA levels. In evaluated patients we did not find anti-ETA Ab, although in 4 cases (6.6%) ETA levels were undetectable. Anti-INF Ab were detected in serum samples from 14 (24.6%) patients, in 13 cases with undetectable serum trough INF levels (Table 4).

At baseline all RA patients had active disease as indicated by a mean (±SD) DAS-28 of 5.76 (±1.35) with no differences in DAS-28 values between patients that subsequently did (3.3 ± 1.55) or did not (3.46 ± 1.73) develop anti-INF or anti-ADA Ab (*P* = 0.727). At baseline all SpA patients had active disease as indicated by a mean (±SD) DAS-28 of 4.48 (±1.19) for peripheral forms, ASDAS 15.41 (±6.13), and BASDAI 5.5 (±2.78) with no differences in those values between patients that subsequently did (2.91 ± 1.32, 5.73 ± 2.1, and 2.2 ± 0.9, resp.) or did not (2.3 ± 1.39, 4.82 ± 3.25, and 2.83 ± 2.31, resp.) develop anti-INF or anti-ADA Ab (*P* = 0.326, *P* = 0.564, and *P* = 0.718, resp.).

**Figure 1:**
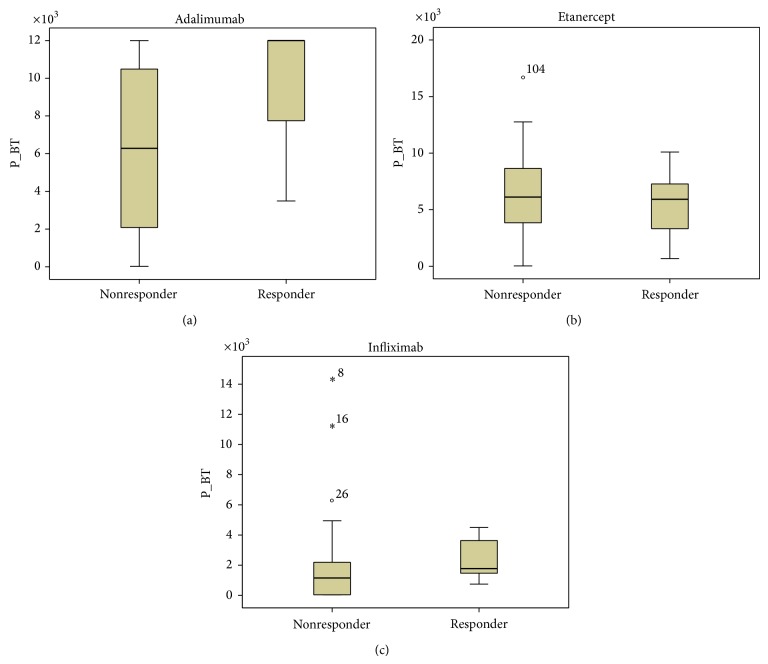
ADA (a), ETA (b), and INF (c) levels (*μ*g/mL) in responders versus nonresponders RA and SpA patients (*P* = 0.142, *P* = 0.488, and *P* = 0.093, resp.). Data presented as interquartile ranges (75th centile, upper edge of the box; 25th centile, lower edge of the box, and 50th centile, midline of the box).

**Figure 2:**
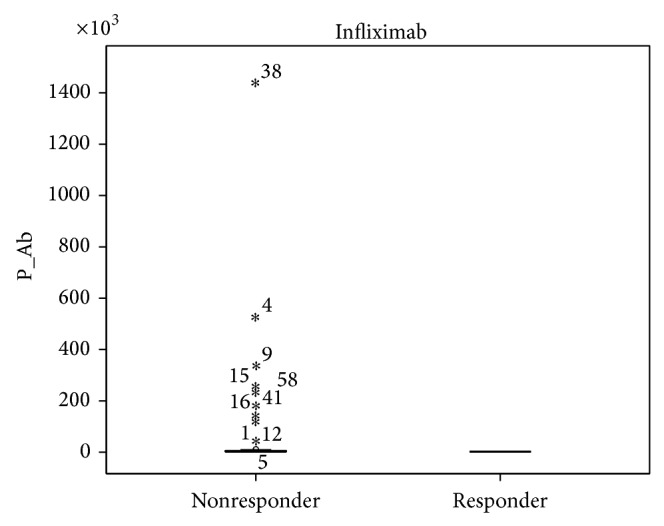
Anti-INF Ab concentration (AU/mL) in responders (*n* = 9) versus nonresponders (*n* = 48) RA and SpA patients (*P* < 0.0001).

**Figure 3:**
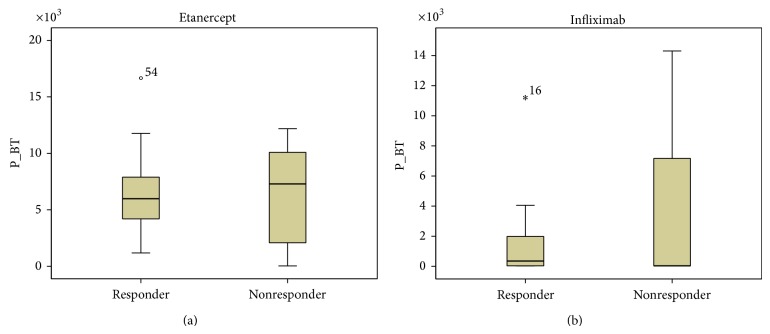
ETA (a) and INF (b) levels (*μ*g/mL) in responders versus nonresponders RA patients (*P* = 0.956 for ETA and *P* = 0.880 for INF). Data presented as interquartile ranges (75th centile, upper edge of the box; 25th centile, lower edge of the box, and 50th centile, midline of the box).

**Figure 4 fig4:**
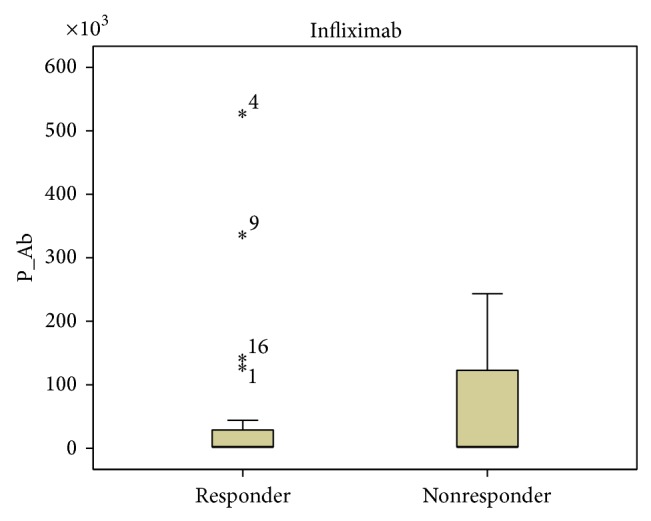
Anti-INF Ab concentration (AU/mL) in responders versus nonresponders RA patients (*P* = 0.956). Data presented as interquartile ranges (75th centile, upper edge of the box; 25th centile, lower edge of the box, and 50th centile, midline of the box).

**Figure 5 fig5:**
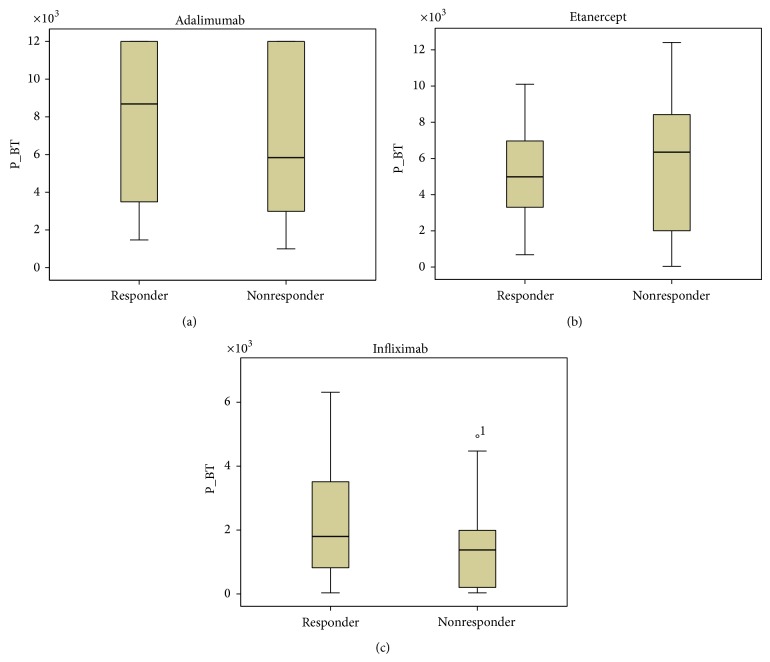
ADA (a), ETA (b), and INF (c) levels (*μ*g/mL) in responders versus nonresponders SpA patients (*P* = 0.861, *P* = 0.618, and *P* = 0.293, resp.). Data presented as interquartile ranges (75th centile, upper edge of the box; and 25th centile, lower edge of the box, 50th centile, midline of the box).

**Figure 6:**
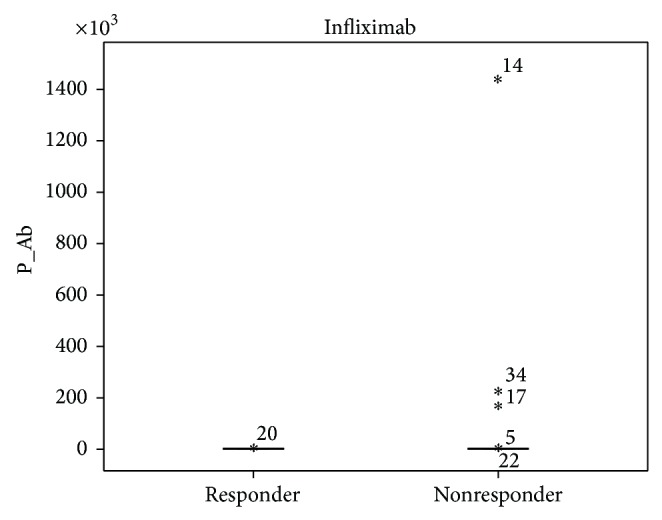
Anti-INF Ab concentration (AU/mL) in responders versus nonresponders SpA patients (*P* = 0.243).

Our results showed a tendency toward higher ADA and INF levels in all patients responding to treatment, but the data was not statistically significant (Figure 1). Patients not responding to treatment had statistically significant higher anti-ADA (*P* < 0.0001) and anti-INF Ab (*P* < 0.0001) concentrations (Figure 2). When analyzing the same data separately in RA and SpA patients results did not differ statistically significant between responders and nonresponders (Figures 3 [Fig fig4] [Fig fig5]–6). All RA and SpA patients, which were responding to treatment, had no detectable anti-drug Ab levels versus nonresponder patients: 1 patient with anti-ADA Ab and 14 patients with anti-INF Ab (*P* < 0.0001).

One RA patient developed anti-ADA Ab (concentration 2000 AU/mL) with no detectable levels of ADA. For this reason ADA was stopped and treatment was changed to rituximab with success. In 4 patients with good treatment response ETA levels were undetectable, although anti-ETA Ab were not found of all ETA treated patients.

We found 14 patients with anti-INF Ab and 13 of them had no detectable levels of INF. Three patients (5,3%) with anti-INF Ab had infusion related reactions, 8 (57.1%) had insufficient treatment effect; 3 patients had good clinical response. In 3 patients (5,3%) with anti-INF Ab, treatment was discontinued, in 3 cases dose was escalated, in 3 biologic drug was changed, and in 5 cases (8,8%) treatment was not changed (patient's decision). In 3 patients (5,3%) INF and anti-INF Ab levels were undetectable.

In order to know the odds of developing infusion related reactions and TNF*α* blockers treatment emendation in patients with anti-INF Ab, we calculated odds ratio (OR) in 143 evaluated patients. Our data shows that patients with anti-INF Ab have higher odds to have infusion related reaction (OR 5.88 (95% CI 1.04–33.28)), to change to another TNF*α* blocker (OR 11.43 (95% CI 1.08–120.93)), to stop treatment with INF (OR 9.28 (95% CI 1.64–52.52)), although 95% CI for these results are wide suggesting low statistical value of these results. Odds to increase INF dose were not statistically significant (OR 2.07 (95% CI 0.43–9.96)). Nevertheless, patients with anti-INF Ab have lower odds of response to treatment (OR 0.8 (95% CI 0.19–3.38), not significant) and to continue INF with the same dose (OR 0.2 (95% CI 0.05–0.69)).

We found negative correlation between MTX use and presence of anti-drug Ab in ADA patients (*Kendall's tau* correlation coefficient −0.686 (*P* = 0.005);* Spearman's rho* correlation coefficient −0.686 (*P* = 0.002)), although in INF patients group correlation was not found (*Kendall's tau* correlation coefficient −0.167 (*P* = 0.220);* Spearman's rho* correlation coefficient −0.142 (*P* = 0.320), resp.).

There was medium negative correlation between INF and anti-INF Ab concentrations (*Kendall's tau* correlation coefficient −0.473 (*P* < 0.0001);* Spearman's rho* correlation coefficient −0.590 (*P* < 0.0001)); low negative correlation between ADA and anti-ADA Ab was found (−0.302 (*P* = 0.088) and −0.348 (*P* = 0.088), resp.).

## 4. Discussion

Our study showed influence of anti-ADA Ab and anti-INF Ab on clinical response and odds to have infusion related reactions or treatment emendation in patients with anti-INF Ab (although it has low statistical value).

In the literature the percentage of patients who develop anti-drug Ab varies among different autoimmune inflammatory diseases. Anti-drug Ab have been seen in up to one third of RA and abour 25% SpA patients [2, 3, 30–32]. Studies have demonstrated that chimeric (mouse-human) drugs, such as INF, have a greater likelihood of inducing anti-drug Ab development than do fully human antibodies [33]. As not all patients treated with anti-TNF agents develop anti-drug Ab, immunogenicity seems to be the result of several factors associated with the treatment, the patient, and the external factors [32]. We have found similar anti-INF Ab formation levels as seen in past studies, 33.3% of patients with RA and 18.2% of patients with SpA. We did not find statistically significant differences between serum TNF*α* blockers concentrations in those responding and not responding to treatment, although previous studies show that serum drug levels strongly correlate with clinical response [2, 3, 6, 19–21, 32]. These results could be explained by small amount of patients in each group and low initial INF doses. However, we have found increased odds in changing treatment from INF to another TNF*α* blocker, stopping treatment or having insufficient treatment effect in patients with anti-INF Abs.

As reported in previous studies patients receiving treatment with INF show a high rate of infusion-related reactions. We found 5,3% patients treated with anti-INF Ab who developed infusion reactions [20]. These data support the view that detectable titers of anti-INF Ab are associated with increased risk of infusion reactions, probably because of the formation of immune complexes and also treatment with low initial INF doses [33]. Our findings indicate that the appearance of anti-INF and anti-ADA Ab is associated with a poor clinical treatment effect, the development of infusion related reactions, and TNF*α* blockers treatment emendation. Detectable levels of TNF*α* blockers in sera of 143 patients did not correlate with clinical response to treatment with one of the TNF*α* blockers (the differences were not found between those who responded or not to the treatment).

In our clinic, for all INF patients, treatment was started with low doses (mean 2.7 (±1.67) mg/kg). Almost half of patients needed dose escalation due to insufficient clinical treatment effect; however only 5.3% of patients with anti-INF Ab dose were escalated due to this reason. This was also shown in previous publications [34–37]. Although patients in our clinic started treatment with lower than recommended INF dosage, the percentage of patients, requiring dose escalation or rate of infusion reactions, is similar as reported in the studies with adequate INF doses [18]. Levels of low TNF*α* blockers concentrations in sera did not correlate with clinical response in our patients. However it seems that low INF trough levels could influence formation of anti-INF Ab in RA and SpA patients. On the contrary, our data on amount of patients with positive anti-INF Ab titers who were treated with low INF doses did not differ from the data of the studies when the adequate dosage of INF were used [30–32, 35].

As known from the literature ETA has the lowest immunogenicity [4, 7–9]. Accordingly, in our study none of the patients were positive for anti-ETA Ab, complementary, ETA serum drug concentrations were not different in patients responding or not responding to the treatment.

We found one RA patient with positive anti-ADA Ab levels and undetectable concentration of ADA who had poor clinical response. Overall, ADA and anti-ADA Ab serum levels did not correlate with clinical response in RA and SpA patients.

Long disease duration and high disease activity (DAS28 5.76 ± 1.35 in RA patients, ASDAS 15.41 ± 6.13 in axial SpA) before treatment with TNF*α* blockers can be the factors also responsible for the fact that we did not find correlation between detectable levels of TNF*α* blockers and anti-drug Abs with clinical efficacy.

Our study had its weaknesses. We had low numbers of patients in groups, and blood samples from the patients were collected in various time intervals (the longest treatment period with INF), and so detection of anti-drug Abs could be influenced by heterogeneity of time periods of treatment.

## 5. Conclusions

Anti-INF Ab are associated with loss in clinical response, an increase incidence of infusion reactions, probable secondary treatment inefficacy, and treatment emendation. The detection of anti-drug Ab could be helpful in order to understand the reason of treatment inefficacy when choosing an appropriate medication. Testing for immunogenicity could become a part of a patient's everyday clinical management.

## Figures and Tables

**Table 1:** Patient's characteristics before initiation of treatment with TNF*α* blockers.

	RA, *n* = 62 (42.9%)	SpA, *n* = 81 (57.1%)
Median disease duration before initiation of TNF*α* blocker, years, median (IQR)	8.0 (4.0–20.0)	6.0 (2.0–11.75)
CRP, mg/L, mean ± SD	31.71 ± 20.86	35.87 ± 23.30
ESR, mm/h, mean ± SD	42.1 ± 25.17	44.6 ± 26.61
DAS-28, mean ± SD	5.76 ± 1.35	Na
HAQ, mean ± SD	1.37 ± 0.78	1.3 ± 0.63
ASDAS, mean ± SD	Na	15.41 ± 6.13^*^
BASDAI, cm, mean ± SD	Na	5.5 ± 2.78^*^
BASFI, cm, mean ± SD	Na	4.78 ± 2.62^*^
MASES index, mean ± SD	Na	4 ± 2
Patient's global VAS, mm, mean ± SD	64.19 ± 21.45	67.66 ± 20.5
Patient's pain VAS, mm, mean ± SD	63.43 ± 22.29	68.97 ± 20.33
Doctor's global VAS, mm, mean ± SD	57.61 ± 18.16	58.32 ± 11.36
Swollen joints, mean ± SD	17 ± 8	10 ± 8
28 swollen joints, mean ± SD	10 ± 7	Na
Tender joints, mean ± SD	22 ± 15	20 ± 14
28 tender joints, mean ± SD	10 ± 9	Na

Notes: data presented mean ± standard deviation (SD) or median and interquartile range (IQR); ^*^axial forms of SpA.

Abbreviations: RA: rheumatoid arthritis, SpA: spondyloarthritis, CRP: C reactive protein, ESR: erythrocyte sedimentation rate, DAS-28: disease activity score in 28 joints, HAQ: Health Assessment Questionnaire, ASDAS: ankylosing spondylitis disease activity score, BASDAI: *bath* ankylosing spondylitis disease activity score, BASFI: *bath* ankylosing spondylitis functional index, MASES: *Maastricht* ankylosing spondylitis entesitis score, VAS: visual analogue scale, and Na: not applicable.

**Table 2:** RA patient's characteristics when serum samples were collected.

Total RA patients, *n* = 62 (42.9%)	
Gender: female, *n* (%)	51 (82.3)
Median age, years, mean ± SD	50.65 ± 13.47
Months of treatment, when blood samples were collected, median (IQR) (interval):	
ADA	6 (3–9) (3–84)
ETA	30 (3–54) (3–66)
INF	15 (12–51) (6–102)
Number of performed tests, *n* (%)	
ADA	9 (14.5)
ETA	29 (46.8)
INF	24 (38.7)
RF positive, *n* (%)	58 (93.5)
ACCP positive, *n* (%)	26 (41.9)
Erosive disease, *n* (%)	62 (100.0)
TJC-60, median (IQR)	4 (2–12)
SJC-60, median (IQR)	3 (1–8)
Doctor's GDA, mm, mean ± SD	26.48 ± 17.8
Patient's GDA, mm, median (IQR)	36.0 (13.0–56.0)
DAS28, mean ± SD	3.44 ± 1.69
SDAI, median (IQR)	12.5 (6.0–24.6)
HAQ, median (IQR)	1.0 (0.373–1.38)
ESR, mm/h, median (IQR)	15 (8–27)
CRP, mg/L, median (IQR)	2.5 (1.2–5.7)
DMARDs use, *n* (%)	50 (80.6)
MTX use, *n* (%)	43 (69.4)
Medium MTX dose, mg/week, mean ± SD	11.22 ± 4.38
GK use, *n* (%)	45 (72.6)
Prednisolone equivalent dose, mg/d, mean ± SD	7.49 ± 3.35

Notes: data presented mean ± standard deviation or median and interquartile range (IQR) or number (*n*) and percent of total number of patients;

Abbreviations: RA: rheumatoid arthritis; IQR: interquartile range; SD: standard deviation, TJC-60: 60 tender joint count, SJC-60: 60 swollen joint count, GDA:global disease activity visual analogue scale (0–100 mm), RF: IgM rheumatoid factor; ACCP: cyclic citrullinated peptide antibody, DAS28: disease activity score in 28 joints; SDAI: simplified disease activity index; HAQ: Health Assessment Questionnaire, ESR: erythrocyte sedimentation rate; CRP: C-reactive protein; DMARDs: disease-modifying antirheumatic drugs (e.g., methotrexate, sulfasalazine, leflunomide, hydrochloroquine, and azathioprine); MTX: methotrexate, and na: data is not applicable.

**Table 3:** SpA patient's characteristics when serum samples were collected.

Total SpA patients, *n* = 81 (57.1%)	
AS patients, *n* = 49	
PsA patients, *n* = 32	
Gender: male, *n* (%)	58 (71.6)
Median age, years, mean ± SD	41.85 ± 11.23
Months of treatment, when blood samples were collected, median (IQR) (interval):	
ADA	6 (3–18) (3–78)
ETA	30 (12–54) (6–72)
INF	54 (21–66) (3–108)
Number of performed tests, *n* (%)	
ADA	16 (19.8)
ETA	32 (39.5)
INF	33 (40.7)
Erosive disease, *n* (%)	43 (53.1)
HLA-B27 positive, *n* (%)	60 (74.1)
Sacroiliitis, *n* (%)	64 (79.0)
TJC-60, median (IQR)	2 (0–4)
SJC-60, median (IQR)	1 (0–2)
Doctor's GDA, mm, mean ± SD	22.45 ± 14.0
Patient's GDA, mm, median (IQR)	21 (8.25–45.75)
DAS28, mean ± SD	2.37 ± 1.36^*^
HAQ, median (IQR)	0.38 (0–0.88)
BASDAI, mm, median (IQR)	2.24 (1.02–4.66)^**^
ASDAS-CRB, mean ± SD (interval)	4.77 ± 3.2
ESR, mm/h, median (IQR)	8 (4–21)
CRP, mg/L, median (IQR)	3.6 (1.05–7.58)
DMARDs use, *n* (%)	66 (81.56)
MTX use, *n* (%)	63 (77.8)
Medium MTX dose, mg/week, mean ± SD	10.93 ± 5.07
GK use, *n* (%)	35 (43.2)
Prednisolone equivalent dose, mg/d, mean ± SD	8.07 ± 5.77

Notes: data presented mean ± standard deviation or median and interquartile range (IQR) or number (*n*) and percent of total number of patients; ^*^peripheral forms of SpA, ^**^axial form of SpA.

Abbreviations: SpA: spondyloarthritis, AS: ankylosing spondylitis, PsA: psoriatic arthritis, IQR: interquartile range; SD: standard deviation, TJC-60: 60 tender joint count, SJC-60: 60 swollen joint count, GDA: global disease activity visual analogue scale (0–100 mm), DAS28: disease activity score in 28 joints; HAQ: Health Assessment Questionnaire, BASDAI: *Bath* ankylosing spondylitis disease activity index (0–100 mm), ESR: erythrocyte sedimentation rate; CRP: C-reactive protein; DMARDs: disease-modifying antirheumatic drugs (e.g., methotrexate, sulfasalazine, leflunomide, hydrochloroquine, and azathioprine); MTX: methotrexate, and na: data is not applicable.

**Table 4:** Detectable serum levels of TNF*α* blockers and anti-drug Abs.

	All, *n* = 143	RA, *n* = 62	SpA, *n* = 81
ADA tests total, *n*	**25**	**9**	**16**
ADA trough levels, patients *n* (%)	25 (100.0)	8 (88.9)	16 (100.0)
ADA concentration, mean ± SD	8.04 ± 4.2	8.24 ± 3.8	7.1 ± 4.48
Anti-ADA Ab positive, patients *n* (%)	1 (4.0)	1 (11.1)	0
Anti-ADA Ab concentration (one sample)	2000	2000	na
ETA tests total, *n*	**61**	**29**	**32**
ETA trough levels, patients *n* (%)	57 (93.4)	28 (96.6)	29 (90.6)
ETA concentration, mean ± SD	6.54 ± 2.34	6.06 ± 1.18	6.83 ± 3.5
Anti-ETA Ab positive, patients *n* (%)	0	0	0
INF tests total, *n*	**57**	**24**	**33**
INF trough levels, patients *n* (%)	41 (71.9)	14 (58.3)	27 (81.8)
INF concentration, median (IQR) (range)	2.36 (1.95–4.26) (1.52–14.3)	3.77 (1.88–9.4) (1.52–14.3)	2.33 (1.96–4.48) (1.69–35.0)
Anti-INF Ab positive, patients *n* (%)	14 (24.6)	8 (33.3)	6 (18.2)
Anti-INF Ab concentration, median (IQR) (range)	130 (7.97–289.9) (4.89–1440)	136.0 (21.17–313.03) (9.2–527)	74.4 (5.4–489) (4.89–1440)

Notes: calculations for drug and anti-drug Abs concentrations was done only for trough serum levels. ADA ≤0.024 *μ*g/mL, ETA and INF concentrations ≤0.035 *μ*g/mL were considered as not detectable for drugs. Anti-ADA Ab ≤3.5 AU/mL, anti-ETA Ab ≤142.0 AU/mL, and anti-INF Ab ≤2.0 AU/mL concentrations were considered as negative.

Abbreviations: SD: standard deviation, na: data is not applicable.

## References

[1] Kneepkens E. L., Krieckaert C. L. M., van der Kleij D. (2014). Lower etanercept levels are associated with high disease activity in ankylosing spondylitis patients at 24 weeks of follow-up. *Annals of the Rheumatic Diseases*.

[2] Kneepkens E. L., Wei J. C.-C., Nurmohamed M. T. (2015). Immunogenicity, adalimumab levels and clinical response in ankylosing spondylitis patients during 24 weeks of follow-up. *Annals of the Rheumatic Diseases*.

[3] de Vries M. K., Wolbink G. J., Stapel S. O. (2007). Inefficacy of infliximab in ankylosing spondylitis is correlated with antibody formation. *Annals of the Rheumatic Diseases*.

[4] Mazilu D., Opriş D., Gainaru C. (2014). Monitoring drug and antidrug levels: a rational approach in rheumatoid arthritis patients treated with biologic agents who experience inadequate response while being on a stable biologic treatment. *BioMed Research International*.

[5] Mok C. C., van der Kleij D., Wolbink G. J. (2013). Drug levels, anti-drug antibodies, and clinical efficacy of the anti-TNF*α* biologics in rheumatic diseases. *Clinical Rheumatology*.

[6] Bartelds G. M., Krieckaert C. L. M., Nurmohamed M. T. (2011). Development of antidrug antibodies against adalimumab and association with disease activity and treatment failure during longterm follow-up. *The Journal of the American Medical Association*.

[7] Hoshino M., Yoshio T., Onishi S., Minota S. (2012). Influence of antibodies against infliximab and etanercept on the treatment effectiveness of these agents in Japanese patients with rheumatoid arthritis. *Modern Rheumatology*.

[8] de Vries M. K., van der Horst-Bruinsma I. E., Nurmohamed M. T. (2009). Immunogenicity does not influence treatment with etanercept in Patients with ankylosing spondylitis. *Annals of the Rheumatic Diseases*.

[9] Dore R. K., Mathews S., Schechtman J. (2007). The immunogenicity, safety, and efficacy of etanercept liquid administered once weekly in patients with rheumatoid arthritis. *Clinical and Experimental Rheumatology*.

[10] Pérez-Guijo V. C., Cravo A. R., Castro M. D. C., Font P., Muñoz-Gomariz E., Collantes-Estevez E. (2007). Increased efficacy of infliximab associated with methotrexate in ankylosing spondylitis. *Joint Bone Spine*.

[11] Mulleman D., Lauféron F., Wendling D. (2011). Infliximab in ankylosing spondylitis: alone or in combination with methotrexate? A pharmacokinetic comparative study. *Arthritis Research and Therapy*.

[12] Breban M., Ravaud P., Claudepierre P. (2008). Maintenance of infliximab treatment in ankylosing spondylitis: results of a one-year randomized controlled trial comparing systematic versus on-demand treatment. *Arthritis and Rheumatism*.

[13] Sampaio-Barros P. D., Costallat L. T. L., Bertolo M. B., Neto J. F. M., Samara A. M. (2000). Methotrexate in the treatment of ankylosing spondylitis. *Scandinavian Journal of Rheumatology*.

[14] Maini R. N., Breedveld F. C., Kalden J. R. (1998). Therapeutic efficacy of multiple intravenous infusions of anti-tumor necrosis factor *α* monoclonal antibody combined with low-dose weekly methotrexate in rheumatoid arthritis. *Arthritis & Rheumatism*.

[15] Lipsky P. E., van der Heijde D. M. F. M., St. Clair E. W. (2000). Infliximab and methotrexate in the treatment of rheumatoid arthritis. *The New England Journal of Medicine*.

[16] St.Clair E. W., Wagner C. L., Fasanmade A. A. (2002). The relationship of serum infliximab concentrations to clinical improvement in rheumatoid arthritis: results from ATTRACT, a multicenter, randomized, double-blind, placebo-controlled trial. *Arthritis and Rheumatism*.

[17] Arora A., Mahajan A., Spurden D., Boyd H., Porter D. (2013). Long-term drug survival of tnf inhibitor therapy in RA patients: a systematic review of European national drug registers. *International Journal of Rheumatology*.

[18] Fabbroni M., Cantarini L., Caso F. (2014). Drug retention rates and treatment discontinuation among anti-TNF-*α* agents in psoriatic arthritis and ankylosing spondylitis in clinical practice. *Mediators of Inflammation*.

[19] Garcês S., Demengeot J., Benito-Garcia E. (2013). The immunogenicity of anti-TNF therapy in immune-mediated inflammatory diseases: a systematic review of the literature with a meta-analysis. *Annals of the Rheumatic Diseases*.

[20] Wolbink G. J., Vis M., Lems W. (2006). Development of antiinfliximab antibodies and relationship to clinical response in patients with rheumatoid arthritis. *Arthritis and Rheumatism*.

[21] Bender N. K., Heilig C. E., Dröll B., Wohlgemuth J., Armbruster F.-P., Heilig B. (2007). Immunogenicity, efficacy and adverse events of adalimumab in RA patients. *Rheumatology International*.

[22] Pan S. M. D., Dehler S., Ciurea A., Hans-Rudolf Z., Gabay C., Finckh A. (2009). Comparison of drug retention rates and causes of drug discontinuation between anti-tumor necrosis factor agents in rheumatoid arthritis. *Arthritis Care and Research*.

[23] Jois R. N., Leeder J., Gibb A. (2006). Low-dose infliximab treatment for ankylosing spondylitis—clinically- and cost-effective. *Rheumatology*.

[24] De La Torre I., Valor L., Nieto J. C., Montoro M., Varreno L. (2014). Minimum effective dosages of anti-TNF in rheumatoid arthritis: a cross-sctional study. *Reumatología Clínica*.

[25] Arštikytė I., Butrimienė I. (2014). Lietuvos reumatinių ligų biologinės terapijos duomenų bazė: 2007–2013 metų veiklos ataskaita. *Vilniaus Reumatologijos Seminarai*.

[26] Putrik P., Ramiro S., Kvien T. K. (2014). Inequities in access to biologic and synthetic DMARDs across 46 European countries. *Annals of the Rheumatic Diseases*.

[27] van Gestel A. M., Prevoo M. L. L., van't Hof M. A., van Rijswijk M. H., van de Putte L. B. A., van Riel P. L. C. M. (1996). Development and validation of the European League Against Rheumatism response criteria for rheumatoid arthritis: comparison with the preliminary american college of rheumatology and the world health organization/international league against rheumatism criteria. *Arthritis & Rheumatism*.

[28] Castillo-Gallego C., Aydin S. Z., Marzo-Ortega H. (2011). Clinical utility of the new ASAS criteria for spondyloarthritis and the disease activity score. *Current Rheumatology Reports*.

[30] Pascual-Salcedo D., Plasencia C., Ramiro S. (2011). Influence of immunogenicity on the efficacy of long-term treatment with infliximab in rheumatoid arthritis. *Rheumatology*.

[31] Plasencia C., Pascual-Salcedo D., García-Carazo S. (2013). The immunogenicity to the first anti-TNF therapy determines the outcome of switching to a second anti-TNF therapy in spondyloarthritis patients. *Arthritis Research and Therapy*.

[32] Plasencia C., Pascual-Salcedo D., Nuño L. (2012). Influence of immunogenicity on the efficacy of longterm treatment of spondyloarthritis with infliximab. *Annals of the Rheumatic Diseases*.

[33] Anderson P. J. (2005). Tumor necrosis factor inhibitors: clinical implications of their different immunogenicity profiles. *Seminars in Arthritis and Rheumatism*.

[34] van der Maas A., Kievit W., van den Bemt B. J. F., van den Hoogen F. H. J., van Riel P. L., den Broeder A. A. (2012). Down-titration and discontinuation of infliximab in rheumatoid arthritis patients with stable low disease activity and stable treatment: an observational cohort study. *Annals of the Rheumatic Diseases*.

[35] Tenga G., Goëb V., Lequerré T. (2011). A 3 mg/kg starting dose of infliximab in active spondyloarthritis resistant to conventional treatments is efficient, safe and lowers costs. *Joint Bone Spine*.

[36] Inman R. D., Maksymowych W. P. (2010). A double-blind, placebo-controlled trial of low dose infliximab in ankylosing spondylitis. *Journal of Rheumatology*.

[37] Mörck B., Pullerits R., Geijer M., Bremell T., Forsblad-D'Elia H. (2013). Infliximab dose reduction sustains the clinical treatment effect in active HLAB27 positive ankylosing spondylitis: a two-year pilot study. *Mediators of Inflammation*.

